# Novel Postoperative Hypofractionated Accelerated Radiation Dose-Painting Approach for Soft Tissue Sarcoma

**DOI:** 10.1016/j.adro.2023.101391

**Published:** 2023-10-31

**Authors:** Matthew Mills, Justin Miller, Casey Liveringhouse, John M. Bryant, Yuki Kawahara, Vladimir Feygelman, Kujtim Latifi, George Yang, Peter A. Johnstone, Arash O. Naghavi

**Affiliations:** a Department of Radiation Oncology, H. Lee Moffitt Cancer Center and Research Institute, Tampa, Florida; b University of South Florida, Morsani College of Medicine, Tampa, Florida

## Abstract

**Purpose:**

Hypofractionated radiation therapy (RT) offers benefits in the treatment of soft tissue sarcomas (STS), including exploitation of the lower α/β, patient convenience, and cost. This study evaluates the acute toxicity of a hypofractionated accelerated RT dose-painting (HARD) approach for postoperative treatment of STS.

**Methods and Materials:**

This is a retrospective review of 53 consecutive patients with STS who underwent resection followed by postoperative RT. Standard postoperative RT dosing for R0/R1/gross disease with sequential boost (50 Gy + 14/16/20 Gy in 32-35 fractions) were replaced with dose-painting, which adapts dose based on risk of disease burden, to 50.4 and 63, 64.4, 70 Gy in 28 fractions, respectively. The first 10 patients were replanned with a sequential boost RT approach and dosimetric indices were compared. Time-to-event outcomes, including local control, regional control, distant control, and overall survival, were estimated with Kaplan-Meier analysis.

**Results:**

Median follow-up was 25.2 months. Most patients had high-grade (59%) STS of the extremity (63%) who underwent resection with either R1 (40%) or close (36%) margins. Four patients experienced grade 3 acute dermatitis which resolved by the 3-month follow-up visit. The 2-year local control, regional control, distant control, and overall survival were 100%, 92%, 68%, and 86%, respectively. Compared with the sequential boost plan, HARD had a significantly lower field size (total V50 Gy; *P* = .002), bone V50 (*P* = .031), and maximum skin dose (*P* = .008). Overall treatment time was decreased by 4 to 7 fractions, which translated to a decrease in estimated average treatment cost of $3056 (range, $2651-$4335; *P* < .001).

**Conclusions:**

In addition to benefits in cost, convenience, and improved biologic effect in STS, HARD regimen offers a safe treatment approach with dosimetric advantages compared with conventional sequential boost, which may translate to improved long-term toxicity.

## Introduction

Soft tissue sarcomas (STS) are a diverse group of tumors that arise from the mesenchymal or connective tissue and account for 1% of all adult malignancies in the United States.^1^ Because of their rarity and heterogeneity, these tumors represent a significant treatment challenge. Although oncologic excision remains the mainstay of treatment for STS, the addition of radiation therapy (RT) is often recommended to reduce the risk of local failure.^2^ Intensity modulated radiation therapy (IMRT), rather than conventional external beam radiation therapy, is commonly used in the postoperative setting after demonstrating significant benefits in local control and avoidance of nearby organs-at-risk (OARs).^3^ Although the total dose and treatment volume depend on clinical factors, conventional RT fractionation of 1.8 to 2 Gy per fraction is most commonly used for STS.

Hypofractionated accelerated RT holds several potential advantages in the treatment of STS. The lower total fractions can improve patient convenience and lower costs,^4^ while also limiting population interactions during the ongoing global pandemic. Additionally, the higher radiation dose per fraction has a higher biologically effective dose (BED) for STS, which has an alpha beta (α/β) ratio of 2 to 6 Gy.^5^, ^6^, ^7^ Because of these potential advantages, there has been recent interest in hypofractionated RT for STS, with numerous studies demonstrating safety and efficacy in the preoperative setting.^8^, ^9^, ^10^, ^11^, ^12^, ^13^, ^14^ The utility of postoperative hypofractionation has been explored with brachytherapy, with local control rates of ∼90% for high-grade STS receiving 30 to 50 Gy over 1 week.^15^ Although this dose is commonly prescribed to a 2 × 1 cm expansion of the tumor bed, there are 125% to 200% isodose lines near the source that simultaneously escalate doses at the highest area of recurrence risk (eg, tumor bed).^16^ In the postoperative setting, outside of brachytherapy, the utilization of hypofractionation with simultaneous dose escalation is not well characterized.

To harness the benefits of hypofractionation while limiting normal tissue toxicity risk, we created a novel accelerated simultaneous integrated boost (SIB) regimen to replace the standard postoperative RT approach in STS (2 Gy per fraction with a cone down sequential boost), which was inspired by the dosimetric advantages of postoperative brachytherapy. This approach, termed “hypofractionated accelerated radiation dose-painting” (HARD), adjusts the dose delivered per day by the volume's clinical risk of disease burden. The low-risk volume is treated with a 50.4 Gy base and a dose-painted volume receiving 63, 64.4, or 70 Gy in 28 fractions for R0, R1, or gross disease, respectively. In this way, the novel HARD technique has the potential to optimize local control (LC) through dose-escalation of the high-risk area while minimizing dose to nearby OARs. The present study evaluates the acute toxicity of this HARD technique, along with the difference in expected long-term toxicity via a dosimetric comparison of the first ten SIB plans to their standard sequential RT boost counterparts.

## Methods and Materials

This is a retrospective review of a prospectively maintained database of 53 patients with STS who underwent resection followed by postoperative RT with the HARD approach, from October 2019 to June 2022, with an accelerated plan of 50.4 Gy as the base target dose and the dose-painted volume receiving 63, 64.4, or 70 Gy in 28 fractions for R0, R1, or gross disease after surgery, respectively. Of note, our practice has standardized postoperative treatment planning (e.g. magnetic resonance imaging, MRI) for patients at high local recurrence risk, to account for gross residual/recurrent disease after surgery, before the start of RT. The dose for postoperative radiation is 63 and 64.4 Gy for negative and positive surgical margins, respectively, unless gross disease was identified on treatment planning imaging. In cases where re-resection was not deemed feasible, gross disease was dose escalated to 70 Gy in 28 fractions. Equivalent dose in 2 Gy per fractions (EQD_2_) was calculated assuming an α/β of 4 to 10.^5^^,^^17^^,^^18^ A computed tomography (CT) simulation was performed with ≤3 mm slices, and immobilization with a vac-lock or aquaplast system was used. Presurgical MRI, when available, was fused to the CT to delineate areas at risk. Gross tumor volume (GTV) was commonly defined by the T1 post contrast, whereas a T2 fat-saturated or STIR image was used to determine extent of initial peritumoral edema. Radio-opaque wires were used to delineate the scar and drain sites. The clinical target volumes (CTV) were defined by risk of microscopic disease, either low (CTV1: 50.4 Gy) or intermediate (CTV2: 63-64.4 Gy). CTV1 was defined as 3 to 4 cm expansion along the muscle/subcutaneous tissue with a 1.5 cm radial expansion from the preoperative GTV and tumor bed, respecting anatomic boundaries (eg, bone, fascia, compartments, organs), including surgically manipulated tissue (eg, scars and drains). CTV2 was a 2 cm by 1 to 1.5 cm expansion from GTV/tumor bed. Residual/recurrent GTV was planned to 70 Gy in 28 fractions (2.5 Gy per fraction). Planning target volume (PTV) was a 3 to 5 mm expansion from CTV or GTV, excluding 3 mm from skin surface if skin was not initially involved. Treatment was planned for PTV V100>95% and minimum point dose (0.03 cc) >95% of the prescribed dose, although V95>95% and 90% minimum dose were allowed to meet organ at risk constraints. All patients were planned using intensity modulated radiation therapy (IMRT) with volumetric modulated arc therapy, and daily CT image-guided radiation therapy. Acute toxicity during and after radiation treatment were reported as per CTCAE (version 5). The present study was approved by the institutional review board of the University of South Florida and Moffitt Cancer Center.

### Dosimetric analysis

Using the same treatment volumes, planning system, dose constraints, and prescription goals, a comparison plan was generated for the first 10 patients with a standard sequential approach with 50 Gy as the base target dose with the boost volume receiving an additional 14, 16, or 20 Gy in 32, 33, or 35 fractions for R0, R1, or gross disease, respectively. The sequential counterpart was planned using the same planning structures, beam energy, beam geometry, treatment planning system, and dose calculation algorithm. A fixed number of iterations were then performed (100) using planning parameters (weighting and cGy values) that were a direct ratio to that of the clinical SIB treatment plan. The HARD regimen and the sequential boost plan counterpart for each patient were then compared for differences in dosimetric indices, including the V40, V50, and maximum dose to the joint and bone, V20, V25, and maximum dose to the skin strip, and the field size (volume receiving ≥50 Gy). Treatment plans were optimized and calculated with the treatment planning system used at the time of patient treatment, including collapsed cone dose calculation in Pinnacle (version 14.6; Phillips), Tomotherapy Phillips ACQ SIM, and Monte Carlo dose engine in Raystation v11A (RaySearch Labratories, Stockholm, Sweden).

### Cost analysis

A sample of patients with STS who received postoperative RT were queried, and the technical fees charged were used to estimate a cost per fraction. The average cost per fraction was then used to extrapolate the cost difference for the HARD regimen of 28 fractions compared with the conventional sequential fractionation regimens of 32, 33, or 35 total fractions.

### Statistics

Descriptive statistics were used to summarize the patient and treatment characteristics of the cohort. Time-to-event outcomes were estimated with Kaplan-Meier analysis from the date of current diagnosis and included LC, regional control, distant control (DC), and overall survival (OS). A local recurrence was defined as a recurrence occurring within high dose PTV (PTV_6300, PTV_6440, PTV_7000), a regional recurrence as outside the high dose PTV but within the 50% isodose line of the low dose PTV (PTV_5040), and distant recurrence as a recurrence beyond the 50% isodose line (eg, lymph node or distant progression, or skip metastases). The Cox proportional hazard model was used for univariate and multivariate analysis to identify significant predictors of DC. Dosimetric variables and estimated treatment costs were compared between the sequential and HARD approaches via the Wilcoxon signed-rank test. The reverse Kaplan-Meier method was used to calculate the median follow-up.^19^ Statistical analyses were performed using JMP 15 (SAS Institute Inc, Cary, NC).

## Results

### Patient and treatment characteristics

Most patients were male (60%) with grade 3 (59%) stage III (45%) STS (Table 1). Most patients received RT to the extremity (62%). There were 19 cases (36%) of close margins and 21 cases (40%) of microscopically positive margins (R1). Thirty-one patients (45%) received a total dose of 63 Gy, 15 patients (19%) received 64.4 Gy, and 7 patients (36%) received 70 Gy (Table 2). Seven patients received preoperative chemotherapy, and 6 patients received postoperative chemotherapy, including 4 patients received concurrent chemoradiotherapy, either ifosfamide (N = 2) or paclitaxel (N = 2).

**Table 1:** Patient and tumor characteristics

Characteristic	No. (%) or median [range]
Age at XRT, y	67.1 [20.2-82.3]
Sex	
Female	21 (39.6%)
Male	32 (60.4%)
KPS	
≥80	47 (88.7%)
60-70	6 (11.3%)
Histology	
Myxofibrosarcoma	9 (17.0%)
Undifferentiated pleomorphic sarcoma	6 (11.3%)
Spindle cell sarcoma	5 (9.4%)
Leiomyosarcoma	4 (7.5%)
Angiosarcoma	3 (5.7%)
Synovial sarcoma	3 (5.7%)
Epithelioid sarcoma	3 (5.7%)
Myxoid liposarcoma	3 (5.7%)
Dedifferentiated liposarcoma	3 (5.7%)
Pleomorphic liposarcoma	2 (3.8%)
Well-differentiated liposarcoma	1 (1.9%)
Extraskeletal osteosarcoma	1 (1.9%)
Extraskeletal myxoid chondrosarcoma	1 (1.9%)
Fibroblastic sarcoma	1 (1.9%)
Malignant peripheral nerve sheath tumor	1 (1.9%)
Myxoinflammatory fibroblastic sarcoma	1 (1.9%)
Pleomorphic dermal sarcoma	1 (1.9%)
Pleomorphic rhabdosarcoma	1 (1.9%)
Sarcoma NOS	4 (7.5%)
Location	
Head and neck	7 (13.2%)
Upper extremity	11 (20.8%)
Lower extremity	22 (41.5%)
Superficial trunk	8 (15.1%)
Deep trunk	5 (9.4%)
Prior recurrence	
Local recurrence	6 (11.3%)
Regional recurrence	2 (3.8%)
Distant recurrence	1 (1.9%)
Clinical stage	
I	3 (5.7%)
II	19 (35.8%)
III	24 (45.3%)
IV	2 (3.8%)
Unknown	5 (9.4%)
Lesion size, cm	5.9 [1.2-19]
Grade	
1	4 (7.5%)
2	13 (24.5%)
3	31 (58.5%)
Unknown	5 (9.4%)

*Abbreviations:* KPS = Karnofsky performance status; NOS = not otherwise specified; XRT = external beam radiation therapy.

**Table 2:** Treatment characteristics

Characteristic	No. (%) or median [range]
Prior unplanned resection	
Yes	18 (34.0)
No	35 (66.0)
Preoperative chemotherapy	
MAI	2 (3.8)
Paclitaxel	2 (3.8)
Doxorubicin/dacarbazine, MAI	1 (1.9)
VAC, vincristine/cyclophosphamide	1 (1.9)
VAC-IE	1 (1.9)
None	46 (86.8)
Postoperative chemotherapy	
Paclitaxel	2 (3.8)
Ifosfamide	2 (3.8)
Doxorubicin/dacarbazine	1 (1.9)
Ifosfamide, vincristine/cyclophosphamide, doxorubicin	1 (1.9)
None	48 (90.6)
Concurrent chemotherapy	
Ifosfamide	2 (3.8)
Paclitaxel	2 (3.8)
None	49 (92.5)
Margin status	
Negative	13 (24.5)
Close	19 (35.8)
Positive	21 (39.6)
Radiation dose, Gy	
70	7 (13.2)
64.4	15 (28.3)
63	31 (58.5)
EQD_2_ (α/β= 4-10), Gy	
72.9-75.8	7 (13.2)
66-67.6	15 (28.3)
64-65.6	31 (58.5)

*Abbreviations:* EQD_2_ = equivalent dose in 2 Gy per fraction; IE = ifosfamide, etoposide; MAI = mesna, doxorubicin, ifosfamide; VAC = vincristine, dactinomycin, cyclophosphamide.

### Clinical outcomes

**Figure 1:**
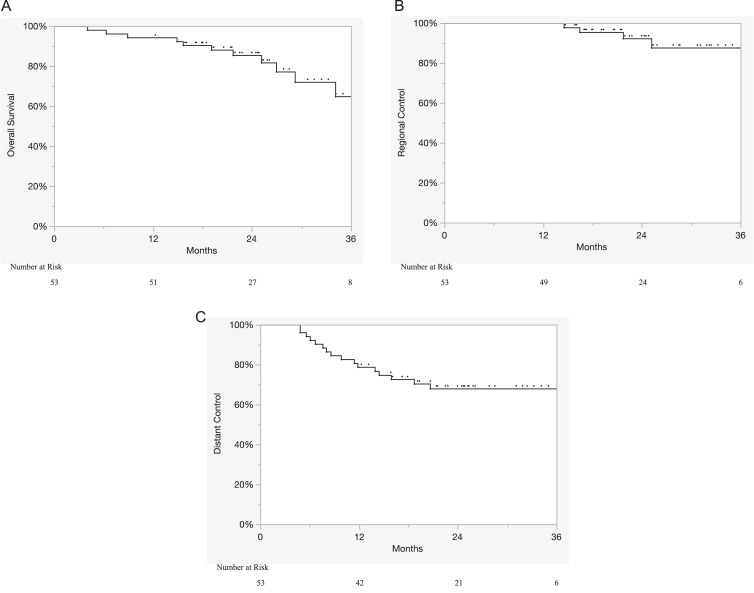
Kaplan-Meier curves depicting (A) overall survival, (B) regional control, and (C) distant control from the date of the current diagnosis.

With a median follow-up of 25.2 months (range, 4-42 months), the 2-year LC, regional control, DC, and OS were 100%, 92.3%, 68.0%, and 85.5%, respectively (Fig. 1A-C). On multivariate analysis, Karnofsky performance status (KPS; hazard ratio [HR], 3.49; *P* = .026) and advanced clinical stage (HR, 7.13; *P* = .01) were significant predictors of inferior DC (Table E1).

Four patients (7.5%) experienced grade 3 toxicities, all of which were acute radiation dermatitis that resolved by the 3-month follow-up visit. All 4 patients who experienced a grade 3 toxicity were either current (N = 1) or former (N = 3) smokers, and no patients with a nonsmoking history experienced grade 3 toxicity. No patients experienced grade 4 or 5 toxicity.

### Dosimetric analysis

**Figure 2:**
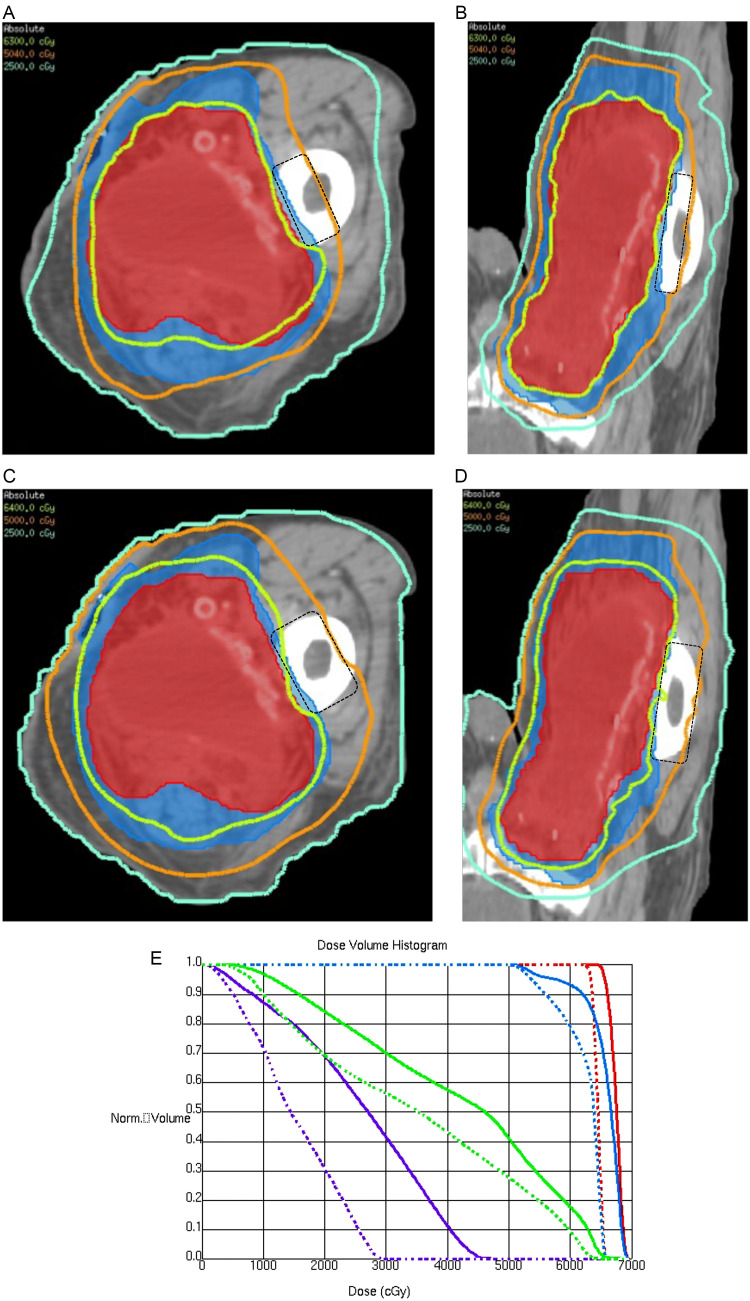
Postoperative radiation plan with isodose lines for a single patient with lower extremity soft tissue sarcoma, treated with the hypofractionated accelerated radiation dose-painting plan to 50.4 Gy in 28 fractions with a simultaneous integrated boost to 63 Gy (A-B), compared with conventional plan to 50 Gy with a sequential boost of 14 Gy (C- D). Note the significantly larger field size (denoted with orange or 50 Gy isodose line) and 50 Gy overlap with bone (black dotted box) in C, D, and E. Dose-volume histogram comparing the hypofractionated accelerated radiation dose plan (solid line) and the conventional plan (dotted line) in high-risk planning target volume (red), low-risk planning target volume (blue), bone (green), and skin strip (purple).

In comparison to the sequential boost plan (Fig. 2), the HARD approach had a statistically significantly lower average field size (1084 cm^3^ vs 1302 cm^3^, *P* = .002), bone V50 (39% vs 49%, *P* = .031), and maximum dose to the skin (41 Gy vs 45 Gy, *P* = .008, Table 3).

**Table 3:** Dosimetric matched paired analysis of HARD versus sequential boost IMRT

Dosimetric parameter	N	Mean difference	95% CI	*P* value
Field size	10	218 cm^3^	116-319 cm^3^	.002
Joint V40	7	–1.3%	–10.1% to 7.5%	.813
Joint V50	7	5.3%	–6.5% to 17.1%	.375
Joint Max	7	5.76 Gy	–1.71 to 13.2 Gy	.156
Bone V40	7	1.0%	–15.5% to 17.6%	.689
Bone V50	7	10.3%	0.10%-20.6%	.031
Bone Max	7	2.29 Gy	4.74-4.10 Gy	.031
Skin strip max	8	4.10 Gy	1.88-6.32 Gy	.008
Spinal cord max	5	4.84 Gy	–0.93 to10.6 Gy	.0625
PTV boost dose V100	10	7.0%	2.3%-12.6%	.010
PTV int dose V100	10	0.1%	–0.04% to 0.3%	.109

*Abbreviations:* CI = confidence interval; IMRT = intensity modulated radiation therapy; Int = intermediate; PTV = planning target volume; SIB = simultaneous integrated boost.

### Cost analysis

The estimated total technical fees for radiation therapy treatment for the conventional 32, 33, and 35 fraction regimens were $20,166, $20,810, and $21,850, respectively, compared with $17,515 for the HARD regimen. The average difference in cost for the cohort was $3056 (range, $2651-$4335, *P* < .001).

## Discussion

Postoperative hypofractionated RT offers practical benefits for patients over conventional fractionation in cost,^4^ convenience, and reduced population interaction during the current global pandemic.^20^ Additionally, the low α/β ratio of STS makes the disease particularly suitable for hypofractionated RT, where the higher dose per fraction provides a higher biologically effective dose to these relatively radioresistant tumors.^5^^,^^7^ Despite these advantages, the role of hypofractionation RT in the postoperative treatment of STS is not well-characterized, and potentially limited to due risk of long-term toxicity.^12^ Herein, we demonstrate the safety and efficacy of an isotoxic postoperative RT approach, using HARD, for risk adapted dosing in STS.

Although there is considerable heterogeneity in STS radiosensitivity, overall they tend to be more radioresistant.^17^^,^^21^, ^22^, ^23^ In a recent clonogenic survival assay analysis of 14 sarcoma cell lines, Haas et al found that the while the median α/β was 4.9 Gy, the radiosensitivity varied considerably by histology, with an α/β of <4 Gy in 6 cell lines.^17^ We recently highlighted the heterogeneity in radiosensitivity within STS in a study using the radiosensitivity index (RSI), a 10-gene signature validated to estimate the intrinsic radiosensitivity of tumors.^22^ The RSI not only confirmed the relative radioresistance of STS, but it also identified a highly radioresistant subset of STS tumors with a lower estimated α/β of 3.29 Gy that may benefit from dose escalation. The higher dose per fraction has a more pronounced effect on tumors with lower α/β but poses a risk to long-term sequela (α/β = 3). In sarcoma, prior studies have described hypofractionated accelerated postoperative RT techniques,^24^, ^25^, ^26^ and others have described dose-painting approach,^27^ but this study is the first to describe the clinical outcomes for a combined hypofractionated accelerated approach with dose-painting technique (“HARD”) for postoperative STS. This technique is a safe targeted BED escalation to the high-risk volumes, with a relatively lower BED to the surrounding normal tissue, thus improving the therapeutic window compared with a standard sequential boost approach.

There is a growing trend toward using preoperative RT, and it is often weighed against the higher risk of wound complications and implications for the patient. In patients with a significant wound healing risk (eg, diabetes, smoking history, superficial/subcutaneous tumors, peripheral vascular disease), or where the difficulty in wound healing recovery outweighs the potential late toxicity benefit with preoperative RT (eg, poor KPS, older age), postoperative RT can be considered. Even in situations best suited for neoadjuvant RT, patients may have a preference for upfront surgery due to tumor related pain, ulceration, or personal choice. Postoperative RT, in comparison to preoperative RT, requires larger treatment fields with higher RT doses, which are associated with an increased risk of long term toxicity (eg, joint stiffness, edema, and fibrosis).^28^^,^^29^ There is concern that hypofractionated RT in the postoperative setting could further increase the risk for late toxicity, as the presumed α/β ratio for normal tissue's late effects is relatively low.^3^, ^4^ Thus, these late effects are more sensitive to higher dose per fraction.^6^^,^^21^ Although the present study lacks the long-term follow-up to adequately address late toxicity concerns, the HARD regimen was associated with significant dosimetric advantages compared with the standard sequential boost plan (Table 3). In particular, the first 10 patients treated with the HARD approach resulted in a smaller 50 Gy field size, compared with their sequential boost counterpart, which is predictive of subcutaneous fibrosis and joint stiffness in the NCIC SR2 trial.^29^ In addition, HARD allowed sparing around weight bearing bones at risk, translating to a significantly lower V50, a known predictor for osteoblast cell death and fracture.^30^ This is consistent with prior dosimetric studies that showed an improved target coverage and reduced OAR doses achieved with postoperative IMRT SIB techniques.^31^^,^^32^ With sarcoma's low α/β, hypofractionation and improving dose conformality (eg, avoiding “cold spots”) are the keys to mitigate risk of recurrence.^3^ After the standard base treatment, the sequential boost's low dose beyond the tumor bed increases the overall field size (50 Gy isodose line) and dose to the adjacent targets, whereas the HARD approach allows a steep dose drop off without the concern of hyperfractionating any of the areas at risk. These dosimetric indices suggest that long-term benefit may be possible when using HARD compared with conventional sequential fractionation.

The HARD regimen was associated with a low risk of acute toxicity, as only 4 patients (7.5%) experienced acute grade 3 radiation dermatitis, which resolved by the 3-month follow-up, and no patients experienced grade 4 or 5 toxicity. In a recent study of 90 patients with STS treated with standard postoperative RT with or without concurrent chemotherapy, Greto et al found higher rates of acute toxicity, with 17% of patients experiencing grade 3 dermatitis.^33^ In the NCIC SR2 trial^28^ comparing preoperative RT and postoperative RT in patients with STS, there was a 68% rate of grade 2 or greater acute skin toxicity for those in the postoperative group, which is consistent with our results.

Five-year local control rates for STS treated with surgery followed by postoperative RT range from 83% to 100%.^22^^,^^28^^,^^34^^,^^35^ With a limited follow-up, our results suggest that the HARD regimen may have similar local control, with no incidences of local failure, and only 4 regional failures (1 within prior PTV_5040, 3 outside of PTV_5040 but within prior 50% isodose line). This is despite the high-risk population in the present study, with a majority of large, grade 3, recurrent tumors with close or positive margins (Table 1). In addition, there were 7 patients with gross disease identified on treatment planning imaging before the start of radiation therapy, where re-resection was not feasible, and included 1 patient with a positive margin resection, 2 patients with gross nodal disease, and 4 patients who developed gross disease at the surgical site after a negative margin resection. Gross disease was dose escalated to 70 Gy without evidence of local recurrence. Longer follow-up is required to confirm the efficacy of the accelerated HARD regimen, although the dose-painting approach has the potential to improve local control with an increased BED while effectively sparing the adjacent organs at risk (Table 3, Fig. 2). Much of our cohort have a high distant recurrence risk (86% stage II-IV), as predicted in previous studies,^36^^,^^37^ which is likely reflective in the 32% 2-year distant recurrence rate observed. Although many patients were at a high distant recurrence risk, 44 of 53 did not receive any chemotherapy, because of patient decision or being deemed a poor chemotherapy candidate by a medical oncologist. Of note, the contributing factors that may have precluded the patient from receiving chemotherapy include patient age (≥70 years, n = 19), prior chemotherapy history (n = 2), KPS ≤70 (n = 6), or comorbidities (eg, chronic kidney disease [n = 5], significant coronary artery disease [n = 10]). On multivariate analysis accounting for size, grade, margins, and gross disease after surgery, we found that only poor KPS and advanced clinic stage were associated with poor DC (Table E1).

The accelerated HARD regimen reduces the total number of fractions from 32-35 to 28, and the difference of 4 to 7 fractions has significant implications not only in patient convenience, but also in health care cost. This difference of 4 to 7 total fractions has an estimated health care cost savings of $2651 to $4335 per patient, in addition to the cost of travel, lodging, childcare, and lost wages incurred for each patient by undergoing an additional week of radiation therapy. Additionally, the HARD regimen offers a potential benefit in reducing exposure to the health care system and potential infection for cancer patients who are often immunocompromised.

There are several important limitations of the present study, including its nonrandomized, retrospective nature from a single institution. The limited follow-up of the present study restricted the ability to assess long-term local control and toxicity. In addition, our results are limited by the significant heterogeneity of the present cohort, especially within STS histology.

## Conclusion

The HARD regimen offers a safe, condensed postoperative RT approach for STS, with similar LC and acute toxicity as historic data. With significant dosimetric benefits, including a lower field size volume and dose to surrounding structures, the utility of HARD may improve long-term toxicity compared with standard postoperative RT with sequential boost, though longer follow-up is required.

## Disclosures

Vladimir Feygelman has served as a consultant for Viewray Inc, received research funding from Varian Medical Systems, and received honoraria from Sun Nuclear Corp and Elekta. Kujtim Latifi has served as a consultant for Viewray Inc. No other disclosures were reported.

## Appendix Supplementary materials

Supplementary Table Supplemental Table 1. Univariate and Multivariate analysis for Distant Control Image, application 1

## Footnotes

Supplementary material associated with this article can be found in the online version at doi:10.1016/j.adro.2023.101391.

## References

[1] Siegel RL, Miller KD, Fuchs HE (2022). Cancer statistics, 2022. CA Cancer J Clin.

[2] Salerno KE, Alektiar KM, Baldini EH (2021). Radiation therapy for treatment of soft tissue sarcoma in adults: Executive summary of an astro clinical practice guideline. Pract Radiat Oncol.

[3] Folkert MR, Singer S, Brennan MF (2014). Comparison of local recurrence with conventional and intensity-modulated radiation therapy for primary soft-tissue sarcomas of the extremity. J Clin Oncol.

[4] Hunter D, Mauldon E, Anderson N (2018). Cost-containment in hypofractionated radiation therapy: A literature review. J Med Radiat Sci.

[5] Yang G, Yuan Z, Ahmed K (2021). Genomic identification of sarcoma radiosensitivity and the clinical implications for radiation dose personalization. Transl Oncol.

[6] Williams MV, Denekamp J, Fowler JF (1985). A review of alpha/beta ratios for experimental tumors: Implications for clinical studies of altered fractionation. Int J Radiat Oncol Biol Phys.

[7] Weichselbaum RR, Beckett MA, Vijayakumar S (1990). Radiobiological characterization of head and neck and sarcoma cells derived from patients prior to radiotherapy. Int J Radiat Oncol Biol Phys.

[8] Leite ETT, Munhoz RR, Camargo VP (2021). Neoadjuvant stereotactic ablative radiotherapy (sabr) for soft tissue sarcomas of the extremities. Radiother Oncol.

[9] Bedi M, Singh R, Charlson JA (2022). Is 5 the new 25? Long-term oncologic outcomes from a phase II, prospective, 5-fraction preoperative radiation therapy trial in patients with localized soft tissue sarcoma. Adv Radiat Oncol.

[10] Kosela-Paterczyk H, Teterycz P, Spalek MJ (2021). Efficacy and safety of hypofractionated preoperative radiotherapy for primary locally advanced soft tissue sarcomas of limbs or trunk wall. Cancers (Basel).

[11] Parsai S, Lawrenz J, Kilpatrick S (2020). Early outcomes of preoperative 5-fraction radiation therapy for soft tissue sarcoma followed by immediate surgical resection. Adv Radiat Oncol.

[12] Valle LF, Bernthal N, Eilber FC (2021). Evaluating thresholds to adopt hypofractionated preoperative radiotherapy as standard of care in sarcoma. Sarcoma.

[13] Guadagnolo BA, Bassett RL, Mitra D (2022). Hypofractionated, 3-week, preoperative radiotherapy for patients with soft tissue sarcomas (HYPORT-STS): A single-centre, open-label, single-arm, phase 2 trial. Lancet Oncol.

[14] Hall KS, Bruland OS, Bjerkehagen B (2020). Preoperative accelerated radiotherapy combined with chemotherapy in a defined cohort of patients with high risk soft tissue sarcoma: A scandinavian sarcoma group study. Clin Sarcoma Res.

[15] Pisters PW, Harrison LB, Leung DH (1996). Long-term results of a prospective randomized trial of adjuvant brachytherapy in soft tissue sarcoma. J Clin Oncol.

[16] Campbell SR, Shah C, Scott JG (2021). American Brachytherapy Society (ABS) consensus statement for soft-tissue sarcoma brachytherapy. Brachytherapy.

[17] Haas RL, Floot BGJ, Scholten AN (2021). Cellular radiosensitivity of soft tissue sarcoma. Radiat Res.

[18] Allignet B, Waissi W, Geets X (2022). Long-term outcomes after definitive radiotherapy with modern techniques for unresectable soft tissue sarcoma. Radiother Oncol.

[19] Xue X, Agalliu I, Kim MY (2017). New methods for estimating follow-up rates in cohort studies. BMC Med Res Methodol.

[20] Spalek MJ, Rutkowski P (2020). Coronavirus disease (covid-19) outbreak: Hypofractionated radiotherapy in soft tissue sarcomas as a valuable option in the environment of limited medical resources and demands for increased protection of patients. Front Oncol.

[21] van Leeuwen CM, Oei AL, Crezee J (2018). The alfa and beta of tumours: A review of parameters of the linear-quadratic model, derived from clinical radiotherapy studies. Radiat Oncol.

[22] Yang JC, Chang AE, Baker AR (1998). Randomized prospective study of the benefit of adjuvant radiation therapy in the treatment of soft tissue sarcomas of the extremity. J Clin Oncol.

[23] Tang Z, Zeng Q, Li Y (2017). Development of a radiosensitivity gene signature for patients with soft tissue sarcoma. Oncotarget.

[24] Domoxoudis S, Koukourakis IM, Giakzidis AG (2019). Hypofractionated accelerated chemo-radiotherapy (chemo-hypoar) with cisplatin and liposomal doxorubicin for the treatment of patients with uterine sarcomas. In Vivo.

[25] Ashby MA, Ago CT, Harmer CL (1986). Hypofractionated radiotherapy for sarcomas. Int J Radiat Oncol Biol Phys.

[26] Heymann S, Jung GM, Simon P (2007). Late outcome of 89 patients with soft-tissue sarcomas treated by surgery and three different radiotherapy schedules [in French]. Cancer Radiother.

[27] Abduljabbar L, Griffin A, Liu Z, Shultz DB (2020). Simulataneous integrated boost during postoperative radiotherapy in extremity soft tissue sarcoma. Int J Radiat Oncol Biol Phys.

[28] O'Sullivan B, Davis AM, Turcotte R (2002). Preoperative versus postoperative radiotherapy in soft-tissue sarcoma of the limbs: A randomised trial. Lancet.

[29] Davis AM, O'Sullivan B, Turcotte R (2005). Late radiation morbidity following randomization to preoperative versus postoperative radiotherapy in extremity soft tissue sarcoma. Radiother Oncol.

[30] Bishop AJ, Zagars GK, Allen PK (2016). Treatment-related fractures after combined modality therapy for soft tissue sarcomas of the proximal lower extremity: Can the risk be mitigated?. Pract Radiat Oncol.

[31] Sladowska A, Hetnal M, Dymek P (2011). Application of imrt in adjuvant treatment of soft tissue sarcomas of the thigh-preliminary results. Rep Pract Oncol Radiother.

[32] Stewart AJ, Lee YK, Saran FH (2009). Comparison of conventional radiotherapy and intensity-modulated radiotherapy for post-operative radiotherapy for primary extremity soft tissue sarcoma. Radiother Oncol.

[33] Greto D, Loi M, Terziani F (2019). A matched cohort study of radio-chemotherapy versus radiotherapy alone in soft tissue sarcoma patients. Radiol Med.

[34] Zagars GK, Ballo MT, Pisters PW (2003). Prognostic factors for patients with localized soft-tissue sarcoma treated with conservation surgery and radiation therapy: An analysis of 1225 patients. Cancer.

[35] Wang J, Song Y, Liu X (2019). Comparison of outcome and toxicity of postoperative intensity-modulated radiation therapy with two-dimensional radiotherapy in patients with soft tissue sarcoma of extremities and trunk. Cancer Med.

[36] Coindre JM, Terrier P, Guillou L (2001). Predictive value of grade for metastasis development in the main histologic types of adult soft tissue sarcomas: A study of 1240 patients from the french federation of cancer centers sarcoma group. Cancer.

[37] Callegaro D, Miceli R, Bonvalot S (2016). Development and external validation of two nomograms to predict overall survival and occurrence of distant metastases in adults after surgical resection of localised soft-tissue sarcomas of the extremities: A retrospective analysis. Lancet Oncol.

