# Supplementary material for: Novel Postoperative Hypofractionated Accelerated Radiation Dose-Painting Approach for Soft Tissue Sarcoma
Source: Adv Radiat Oncol. 2023 Oct 31;9(3):101391. doi: 10.1016/j.adro.2023.101391 (PMC10943519; doi:10.1016/j.adro.2023.101391)
Supplement: Supplementary Table — Supplemental Table 1. Univariate and Multivariate analysis for Distant Control [file mmc1.docx]

| **Supplemental Table 1.** Univariate and Multivariate analysis for Distant Control | | | | |
| --- | --- | --- | --- | --- |
|  | Univariate | | Multivariate | |
| **Variable** | **HR (95% CI)** | ***P* value** | **HR (95% CI)** | ***P* value** |
| Age at treatment | 1.01 (0.97-1.06) | 0.604 |  |  |
| Karnofsky Performance Status |  |  |  |  |
| 90-100 | 1.00 (Reference) |  | 1.00 (Reference) |  |
| 60-80 | 3.10 (1.15-8.36) | **0.025** | 3.49 (1.16-10.5) | **0.026** |
| Location |  |  |  |  |
| lower extremity | 1.00 (Reference) |  |  |  |
| head and neck | 1.73 (0.43-6.94) | 0.438 |  |  |
| upper extremity | 0.29 (0.03-2.37) | 0.246 |  |  |
| superficial trunk | 1.35 (0.34-5.41) | 0.669 |  |  |
| deep trunk | 2.74 (0.68-11.0) | 0.156 |  |  |
| Margin Status |  |  |  |  |
| Negative | 1.00 (Reference) |  |  |  |
| Close | 0.82 (0.25-2.69) | 0.746 |  |  |
| Positive | 0.58 (0.17-2.01) | 0.390 |  |  |
| Clinical Stage |  |  |  |  |
| I-II | 1.00 (Reference) |  | 1.00 (Reference) |  |
| III-IV | 7.24 (1.63-32.2) | **0.009** | 7.13 (1.59-32.0) | **0.010** |
| Prior unplanned resection |  |  |  |  |
| No | 1.00 (Reference) |  |  |  |
| Yes | 0.55 (0.18-1.71) | 0.303 |  |  |
| Lesion size (maximum diameter) | 1.09 (0.97-1.22) | 0.108 |  |  |
| Tumor grade |  |  |  |  |
| 1-2 | 1.00 (Reference) |  |  |  |
| 3 | 1.90 (0.61-5.90) | 0.266 |  |  |
| Gross disease after surgery |  |  |  |  |
| Gross disease (+) | 1.00 (Reference) |  | 1.00 (Reference) |  |
| Gross disease (-) | 0.23 (0.08-0.68) | **0.008** | 0.34 (0.09-1.25) | 0.104 |
| Any chemotherapy |  |  |  |  |
| No | 1.00 (Reference) |  |  |  |
| Yes | 2.21 (0.76-6.38) | 0.144 |  |  |
| *Abbreviations: HR= hazard ratio; CI = confidence interval.* | | | | |
